# Half-molar sodium lactate infusion improves cardiac performance in acute heart failure: a pilot randomised controlled clinical trial

**DOI:** 10.1186/cc13793

**Published:** 2014-03-25

**Authors:** Marek Nalos, Xavier Maurice Leverve, Stephen Joseph Huang, Leonie Weisbrodt, Ray Parkin, Ian Mark Seppelt, Iris Ting, Anthony Stuart Mclean

**Affiliations:** 1 Department of Intensive Care Medicine, Sydney Medical School–Nepean, University of Sydney, Nepean Hospital, Penrith NSW 2750, Australia; 2 LBFA, INSERM-U884, Université Joseph-Fourier Grenoble, BP 53 X 38041 Grenoble, cedex 9, France

## Abstract

**Introduction:**

Acute heart failure (AHF) is characterized by inadequate cardiac output (CO), congestive symptoms, poor peripheral perfusion and end-organ dysfunction. Treatment often includes a combination of diuretics, oxygen, positive pressure ventilation, inotropes and vasodilators or vasopressors. Lactate is a marker of illness severity but is also an important metabolic substrate for the myocardium at rest and during stress. We tested the effects of half-molar sodium lactate infusion on cardiac performance in AHF.

**Methods:**

We conducted a prospective, randomised, controlled, open-label, pilot clinical trial in 40 patients fulfilling two of the following three criteria for AHF: (1) left ventricular ejection fraction <40%, (2) acute pulmonary oedema or respiratory failure of predominantly cardiac origin requiring mechanical ventilation and (3) currently receiving vasopressor and/or inotropic support. Patients in the intervention group received a 3 ml/kg bolus of half-molar sodium lactate over the course of 15 minutes followed by 1 ml/kg/h continuous infusion for 24 hours. The control group received only a 3 ml/kg bolus of Hartmann’s solution without continuous infusion. The primary outcome was CO assessed by transthoracic echocardiography 24 hours after randomisation. Secondary outcomes included a measure of right ventricular systolic function (tricuspid annular plane systolic excursion (TAPSE)), acid-base balance, electrolyte and organ function parameters, along with length of stay and mortality.

**Results:**

The infusion of half-molar sodium lactate increased (mean ± SD) CO from 4.05 ± 1.37 L/min to 5.49 ± 1.9 L/min (*P* < 0.01) and TAPSE from 14.7 ± 5.5 mm to 18.3 ± 7 mm (*P* = 0.02). Plasma sodium and pH increased (136 ± 4 to 146 ± 6 and 7.40 ± 0.06 to 7.53 ± 0.03, respectively; both *P* < 0.01), but potassium, chloride and phosphate levels decreased. There were no significant differences in the need for vasoactive therapy, respiratory support, renal or liver function tests, duration of ICU and hospital stay or 28- and 90-day mortality.

**Conclusions:**

Infusion of half-molar sodium lactate improved cardiac performance and led to metabolic alkalosis in AHF patients without any detrimental effects on organ function.

**Trial registration:**

Clinicaltrials.gov NCT01981655. Registered 13 August 2013.

## Introduction

Critically ill patients develop acute heart failure (AHF) *de novo* or experience exacerbation of chronic heart failure due to coronary ischaemia, arrhythmias, medication noncompliance, worsening renal function, primary infection or other illness [1]. Symptoms are usually related to fluid overload and poor peripheral perfusion, leading to end-organ dysfunction [2]. Standard treatment options include oxygen, positive pressure ventilation, diuretics, vasodilators, inotropes and vasopressors, despite their potential disadvantages and side effects [3]. Vasodilators are associated with tachyphylaxis and hypotension, and inotropes and vasopressors cause tachycardia, arrhythmias, coronary ischaemia and worse overall survival in AHF patients [4]. The promising inotropic agent levosimendan has not convincingly led to superior outcomes [5-7]. There is a clear need for AHF treatment that is noninvasive, increases myocardial contraction by a mechanism of action independent of cAMP activation and has the potential to improve organ function and patient outcomes [8].

Blood lactate is regarded as a marker of illness severity, and increased plasma lactate levels are common in AHF and acute coronary syndromes [9-11]. Although some evidence derived from animal experiments suggests that lactate has direct negative effects on myocardium [12], data reported more recently support the role of lactate as a preferred oxidative substrate in stressed myocardium [13-16]. Overall lactate metabolism in severe cardiogenic shock has been found not to be significantly altered, and half-molar sodium lactate infusion increased cardiac output (CO) while a negative fluid balance was maintained in patients after coronary artery bypass grafting [17,18].

Herein we report the results of our prospective, open-label, pilot, randomised, controlled clinical trial of half-molar sodium lactate infusion to improve cardiac function in critically ill patients with acute heart failure and reduced left ventricular ejection fraction (LVEF).

## Methods

The study was conducted between December 2009 and November 2012 in the Department of Intensive Care Medicine at Nepean Hospital, University of Sydney, Australia. The Sydney West Area Health Service Ethics Committee approved the study (HREC/09/NEPEAN/4) in accordance with the 1964 Declaration of Helsinki and its later amendments. Written informed consent was obtained from the patients or their next of kin prior to enrolment. Adult patients over 18 years of age were prospectively screened and considered eligible if two of three criteria for AHF were met: (1) LVEF ≤40%; (2) acute pulmonary oedema or respiratory failure of predominantly cardiac origin requiring mechanical ventilation, including noninvasive ventilation; and (3) currently receiving inotropes and/or vasopressors. The exclusion criteria were pregnancy, hypernatremia >145 mmol/L, hypertrophic obstructive cardiomyopathy, uncorrected severe valvular heart disease, third-degree heart block, sustained ventricular tachycardia, cardiac tamponade, septic shock, acute respiratory distress syndrome, moribund patients, presence of major diseases with a poor prognosis (such as end-stage cancer, end-stage liver failure (Child-Pugh score III or IV), end-stage (dialysis-dependent) renal failure, and likely to receive haemodialysis and/or filtration within 6 hours of enrolment) and acute liver failure (defined as international normalised ratio of 3) unrelated to warfarin use.

### Study protocol

Following study enrolment, patients were randomised to receive either half-molar sodium lactate (Kalsolac; Aguettant Santé SA, Lyon, France), denominated the lactate group, or Hartmann’s solution (Compound Sodium Lactate Solution; Baxter Australia, Old Toongabbie NSW, Australia), classified as the control group. Randomisation was carried out in blocks of variable size in sequentially numbered, opaque, sealed envelopes provided by an independent statistician. After baseline parameters were recorded and transthoracic echocardiography (TTE) was performed, patients were given an intravenous bolus of 3 ml/kg actual body weight (ABW) of the assigned fluid according to randomisation over the course of 15 minutes. A continuous infusion of half-molar sodium lactate at 1 ml/kg/h ABW for 24 hours was then infused in the lactate group. To prevent potentially harmful fluid overload, no additional study fluid was infused in the control group. The Scientific Committee of the local Ethics Committee mandated this aspect of the protocol. Both groups received standard therapy for AHF according to best practice, which included a combination of inotropes, vasopressors, vasodilators, diuretics, intraaortic balloon counterpulsation and mechanical ventilation as required. Patients had TTE performed 24 and 48 hours after enrolment, except for those who died or were discharged from the ICU. Arterial blood gas analysis was performed at baseline, after the initial study fluid bolus and together with other laboratory and vital sign parameters at 6, 12, 24 and 48 hours after enrolment. These laboratory examinations included fluid balance, electrolytes, full blood count and renal and liver function tests. All patients were followed up for up to 90 days.

### Echocardiography

Qualified sonographers or clinicians performed TTE using ultrasound systems equipped with tissue Doppler (Vivid i, Vivid q or Vivid 7; GE Healthcare, Oslo, Norway). LVEF was estimated on the basis of apical two- and four-chamber views according to Simpson’s formula as recommended by the American Society of Echocardiography [19]. CO was obtained by multiplication of stroke volume (SV) by heart rate (HR) in the left ventricular outflow tract (LVOT) using the Doppler method. SV was determined as follows: π × (LVOT diameter ÷ 2)^2^ × Velocity time integral (VTI) of the blood flow signal in the LVOT. The LVOT diameter was obtained from the parasternal long axis, and the LVOT VTI was determined from the apical five-chamber view using pulsed wave Doppler (PWD). Tricuspid annular plane systolic excursion (TAPSE) in the apical four-chamber view was recorded to assess right ventricular systolic function. Early mitral inflow velocity (*E*) was measured by PWD at the tip of the mitral valve during diastole, and tissue Doppler imaging–derived early diastolic motion velocity (*E*′) and systolic motion of the mitral annulus (averaged medial and lateral) by tissue Doppler imaging (SmTDI) were obtained by placing the sample gate at the level of the mitral annulus of the interventricular septum and lateral wall in the apical four-chamber view. *E*′ and SmTDI from both locations were averaged as recommended previously [20]. A qualified cardiologist and intensivist (RP), who was blinded to treatment assignment, reviewed and measured offline all TTE parameters as appropriate. Intra- and interobserver variability from a random sample of ten patients were calculated.

### Endpoints

The primary study outcome was CO at 24 hours after study entry, which was measured by TTE as described above. Secondary outcome parameters included SmTDI, *E*/*E*′ ratio and TAPSE, overall fluid balance, blood pressure, acid-base status, electrolytes, renal and liver function tests, need for mechanical ventilation (including noninvasive), ICU and hospital lengths of stay and 28- and 90-day mortality.

### Statistical analysis

Data are expressed as mean ± standard deviation (SD). Between-group and within-group differences due to treatment (lactate vs. control) were assessed by two-way repeated-measures analysis of variance and Fisher’s exact test for qualitative data (that is, number of additional treatments used). In cases where significant intra- or intergroup differences were present, pairwise *post hoc* analyses were performed by using Student’s *t*-test. The significance level was set at 0.05 and adjusted for multiple comparisons by the Bonferroni correction method. To adjust for baseline imbalances and the unequal amount of intravenous fluid in the study groups, the ‘regression to the mean’ effects and analysis of covariance (ANCOVA) were carried out using a linear model containing primary outcome as the dependent variable, with treatment as a dummy variable with baseline CO and 24-hour fluid balance used as the covariates. Time to events data were analysed using Kaplan-Meier statistics with survival compared using a logrank test. This trial was a pilot study, so no formal sample size calculation was possible. An arbitrary number of 40 patients was chosen.

## Results

**Figure 1:**
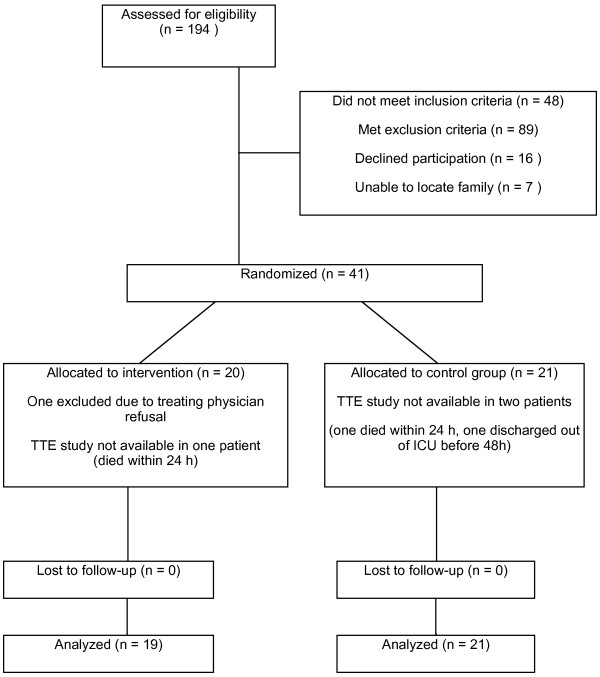
**Flowchart illustrating study protocol for selecting and randomising patients.** The number of screened, excluded, randomised and analysed patients are given.

From among the 194 screened patients, 41 were randomised (Figure 1). One patient randomised to the lactate group was excluded from the analysis because the treating physician deemed the patient unsuitable prior to any intervention. The analysis thus included 40 patients: 19 in the lactate group and 21 in the control group. The baseline patient characteristics are presented in Table 1. Echocardiograms for all three time points were not available for one patient in the lactate group and for two patients in the control group, and thus these patients were excluded from further analysis of echocardiographic parameters.

**Table 1:** **Baseline demographic variables, aetiology of acute heart failure and related chronic premorbid conditions**^**a**^

**Patient characteristics**	**Control group**	**Lactate group**
Age (years)	69.9 ± 9.8	67.4 ± 14.6
Sex (male/female)	17/4	13/6
Weight (kg)	86.5 ± 19.6	79.2 ± 22.3
APACHE II score	18.5 ± 6.9	18.6 ± 5.3
LVEF (%)	27.1 ± 10.3	27.2 ± 8.1
Bilirubin (μmol/L)	15 ± 13	20 ± 25
ALT (IU/L)	489 ± 1,026	525 ± 928
Aetiology of AHF (*n*)^b^		
ADHF	4	2
NSTEMI	8	8
STEMI	5	5
Arrhythmia	1	1
Infection	6	4
Cardiac arrest	1	4
Other	1	1
Premorbid conditions (*n*)^b^		
IHD	14	11
CCF	10	6
HT	10	8
Diabetes	9	5

^a^ADHF = Acute decompensated heart failure; ALT = Alanine aminotransferase; APACHE II = Acute Physiology and Chronic Health Evaluation II; CCF = Chronic congestive heart failure; HT = Hypertension; IHD = Ischaemic heart disease; LVEF = Left ventricular ejection fraction; NSTEMI = Non-ST-segment elevation myocardial infarct; STEMI = ST-segment elevation myocardial infarct. ^b^Some patients had more than one condition present. Data are expressed as mean ± SD unless indicated otherwise.

### Haemodynamics

**Figure 2:**
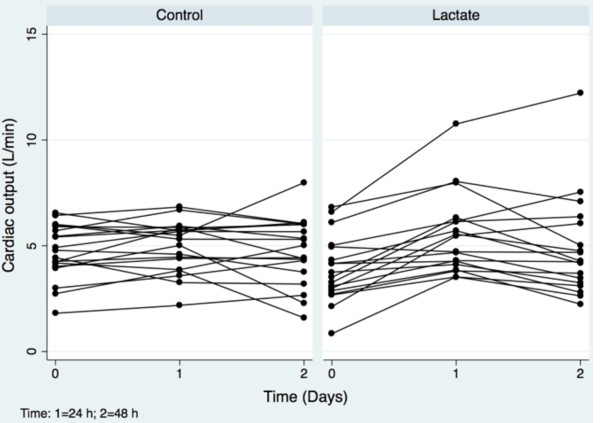
Graphs depict the temporal changes in individual patients’ cardiac output over the course of 24 and 48 hours.

The mean arterial blood pressure remained unchanged throughout the study period in both groups (Table 2). The mean CO was lower in the lactate group at baseline (4.05 ± 1.37 L/min vs. 4.72 ± 1.3 L/min), but this difference was not significant. There was no change in mean CO over time in the control group (4.96 ± 1.21 L/min at 24 hours and 4.76 ± 1.58 L/min at 48 hours). At the end of the 24-hour period, CO significantly increased in the lactate group (5.49 ± 1.9 L/min; *P* < 0.01) and remained higher than at baseline at 48 hours (4.87 ± 2.38 L/min; *P* = 0.026). The improvement in CO remained significant even after adjusting for the baseline imbalance by ANCOVA (Table 3). The temporal change in individual CO over time is presented in Figure 2. The intra- and interobserver variability of CO measurements were 5.9% and 7.2%, respectively. When the components of CO were analysed separately, SV changes, but not HR changes, were responsible for the increase in CO. Similarly, the right ventricular systolic function (TAPSE) was unchanged in the control group but improved in the lactate group (*P* = 0.02) (Table 2). The SmTDI and *E*′ values were not significantly different at any time point in either group, although there was a trend toward increased SmTDI in the lactate group at 24 hours. Although *E*/*E*′ was unchanged over 24 hours in the lactate group (14.4 ± 6.8 to 15.2 ± 7.6), it was reduced (17.3 ± 8.3 to 13.5 ± 4.7; *P* = 0.03) in the control group, suggesting reduction in left atrial pressure (Table 3). There were no significant intergroup differences in the concomitant use of vasoactive and inotropic therapy or in the use of intraaortic balloon counterpulsation, although fewer patients in the lactate group required vasopressor support at 24 hours. More patients were invasively ventilated in the lactate group than in the control group (Table 4).

**Table 2:** **Evolution of haemodynamic and selected transthoracic echocardiography parameters during the study period**^**a**^

**Time points**	**MAP**	**HR**	**SV**	**TAPSE**	**SmTDI**	***E*/ *E *** **′**
Baseline						
Control	78 ± 15	97 ± 23	50.6 ± 13.7	16.3 ± 5	6.0 ± 0.02	17.3 ± 8.3
Lactate	77 ± 16	95 ± 24	49 ± 19.4	14.7 ± 5.5	5.84 ± 0.02	14.4 ± 6.8
24 hours						
Control	78 ± 12	91 ± 14	53.3 ± 13.5	16 ± 5.5	6.53 ± 0.02	13.5 ± 4.7^a^
Lactate	79 ± 17	95 ± 15	59.6 ± 20^b^	18.3 ± 7^b^	7.0 ± 0.04	15.2 ± 7.6
48 hours						
Control	77 ± 15	91 ± 15	52.3 ± 16	17.1 ± 5.7	6.5 ± 0.02	13.8 ± 5.7^a^
Lactate	85 ± 18	89 ± 17	54.1 ± 26.5	18.1 ± 7.8^a^	6.2 ± 0.03	15.9 ± 9.7

^a^*E*/*E′* = Ratio of early peak diastolic transmitral Doppler flow velocity to early peak diastolic tissue Doppler velocity of the mitral annulus (averaged medial and lateral); HR = heart rate (beats/min); MAP = Mean arterial pressure (mmHg); SmTDI = Systolic motion of the mitral annulus (averaged medial and lateral) by tissue Doppler imaging (cm/s); SV = Stroke volume; TAPSE = Tricuspid annular plane systolic excursion (mm). ^b^*P* < 0.05 and ^c^*P* < 0.01 compared to baseline values. Data are expressed as mean ± SD.

**Table 3:** **Analysis of covariance data**^**a**^

**Covariate**	**β**	**SE**	**95% CI**	***P*-value**
Constant	0.79	0.86	-0.97 to 2.55	0.365
Treatment	0.91	0.34	0.22 to 1.57	0.011
Baseline CO	0.71	0.12	0.47 to 0.95	<0.001
Fluid balance	-0.000047	0.00097	-0.00025 to 0.00015	0.627

^a^β: Regression coefficient; CI: Confidence interval; CO: Cardiac output. *F*-value for the model = 12.12. P < 0.001.

**Table 4:** **Evolution of concomitant treatment and interventions during the study period**^**a**^

**Time points**	**Intervention**	**Control**	**Lactate**
Baseline	Vasopressors	9	9
	Vasopressor dose (μg/kg/min)	0.04 ± 0.06	0.07 ± 0.1
	Levosimendan (0.1 μg/kg/min)	5	4
	Dobutamine	1	0
	IABP	2	4
	Invasive ventilation	10	14
	Noninvasive ventilation	5	1
24 hours	Vasopressors	7	3
	Vasopressor dose (μg/kg/min)	0.03 ± 0.05	0.03 ± 0.1
	IABP	2	4
	Invasive ventilation	9	10
	Noninvasive ventilation	5	0
48 hours	Vasopressors	2	3
	Vasopressor dose (μg/kg/min)	0.01 ± 0.03	0.04 ± 0.1
	IABP	2	3
	Invasive ventilation	4	9
	Noninvasive ventilation	1	2

^a^IABP = Intraaortic balloon counterpulsation device. Vasopressors are noradrenaline or adrenaline. Levosimendan (12.5 mg) was administered at 0.1 μg/kg/min over the course of 24 hours during the study period. Infusions were always started prior to study enrolment. All data are expressed as number of patients, except for vasopressor dose, for which data are expressed as mean ± SD.

### Acid-base status

There were no significant changes in any of the acid-base parameters in the control group apart from significant increases in bicarbonate levels and base excess (BE) at 48 hours. In the lactate group, pH increased from 7.4 ± 0.06 to 7.45 ± 0.05, bicarbonate from 23.3 ± 3.3 to 25.9 ± 4.1 mmol/L, BE from -0.3 ± 4.1 to 2.7 ± 4.2 mmol/L and lactate from 2.4 ± 2.3 to 4.8 ± 2.0 mmol/L (all *P* < 0.01) within 15 minutes of half-molar sodium lactate solution bolus. The pH, bicarbonate and BE continued to rise over the 24-hour period (all *P* < 0.01). Once the half-molar sodium lactate infusion ceased, pH, bicarbonate and BE declined, but they remained higher than either baseline or the control group at 48 hours. Plasma lactate remained elevated during the infusion; however, the mean lactate at 24 hours was similar to baseline and declined further, so that it was lower than in the control group at 48 hours (Table 5).

**Table 5:** **Evolution of acid base parameters in arterial blood during the study period**^**a**^

**Time point**	**pH**	**PaCO**_**2**_	**PaO**_**2**_	**HCO**_**3**_^**-**^	**BE**	**Lactate**
Baseline						
Control (*n* = 21)	7.37 ± 0.1	40 ± 13	98 ± 33	22.8 ± 4.9	-1.7 ± 5.6	1.4 ± 0.5
Lactate (*n* = 19)	7.40 ± 0.06	36 ± 9	103 ± 28	23.3 ± 3.3	-0.3 ± 4.1	2.4 ± 2.3
Post-bolus						
Control (*n* = 21)	7.37 ± 0.09	40 ± 9	91 ± 19	23.1 ± 5.1	-1.01 ± 5.7	2 ± 1.4
Lactate (*n* = 19)	7.45 ± 0.05^b,c^	38 ± 8	95 ± 30	25.9 ± 4.1^b^	2.7 ± 4.2	4.8 ± 2.0^b^
6 hours						
Control (*n* = 20)	7.38 ± 0.06	42 ± 9	93 ± 27	24.1 ± 4.8	-0.7 ± 5.3	1.6 ± 1.2
Lactate (*n* = 19)	7.49 ± 0.04^b,c^	43 ± 5^b^	87 ± 24	32.5 ± 4.8^b,c^	8.9 ± 4.5^b,c^	2.9 ± 1.0^c^
12 hours						
Control (*n* = 19)	7.38 ± 0.07	42 ± 10	95 ± 37	24.3 ± 4.7	-0.2 ± 5.2	1.4 ± 0.6
Lactate (*n* = 19)	7.51 ± 0.04^b,c^	47 ± 5^b^	82 ± 24	37.1 ± 5.3^b,c^	12.9 ± 4.6	3.1 ± 1.6^c^
24 hours						
Control (*n* = 19)	7.41 ± 0.07	38 ± 11	84 ± 20	23.9 ± 5.4	-0.9 ± 5.8	1.5 ± 1.0
Lactate (*n* = 18)	7.53 ± 0.03^b,c^	51 ± 7^b,c^	81 ± 26	41.4 ± 6.3^b,c^	16.8 ± 4.8^b,c^	2.3 ± 0.8^c^
48 hours						
Control (*n* = 18)	7.42 ± 0.03	42 ± 9	87 ± 28	26.9 ± 5.6^b^	2.8 ± 5.3^b^	1.6 ± 0.9
Lactate (*n* = 18)	7.47 ± 0.03^b^	48 ± 4^b^	95 ± 42	34.2 ± 4.1^b,c^	9.6 ± 4.2^b,c^	1.2 ± 0.5^b^

^a^BE = Base excess (mmol/L); HCO_3_^-^ **=** Bicarbonate (mmol/L); PaCO_2_ = Partial pressure of carbon dioxide (mmHg); PaO_2_ = Partial pressure of oxygen (mmHg). ^b^*P* < 0.01 compared to baseline values and ^c^*P* < 0.01 for intergroup comparison. Data are expressed as mean ± SD.

### Electrolytes

There were significant changes in electrolyte levels in the lactate group. The sodium level increased from 136 ± 4 mmol/L at baseline to 146 ± 6 mmol/L at 24 hours (*P* < 0.01). We also noted a significant decrease in levels of potassium (4.2 ± 0.7 to 3.3 ± 0.4 mmol/L; *P* < 0.01), chloride (104 ± 7 to 98 ± 9 mmol/L; *P* < 0.01) and phosphate (1.23 ± 0.46 to 0.98 ± 0.40 mmol/L; *P* = 0.04), despite clinically driven supplementation. There were no significant changes in electrolyte levels in the control group (Table 6).

**Table 6:** Evolution of biochemistry parameters during the study period

**Time point**	**Na**^**+**^	**K**^**+**^	**Cl-**	**P**	**Crea**
Baseline					
Control (*n* = 21)	137 ± 5	4.3 ± 0.8	104 ± 5	1.46 ± 0.62	175 ± 107
Lactate (*n* = 19)	136 ± 4	4.2 ± 0.7	104 ± 7	1.23 ± 0.46	146 ± 123
6 hours					
Control (*n* = 20)	137 ± 5	4.2 ± 0.6	104 ± 4	1.36 ± 0.54	174 ± 124
Lactate (*n* = 19)	141 ± 5^b,c^	3.8 ± 0.4^d^	100 ± 7^b^	0.95 ± 0.50^b^	138 ± 126
12 hours					
Control (*n* = 19)	137 ± 6	4.3 ± 0.6	104 ± 5	1.40 ± 0.58	186 ± 135
Lactate (*n* = 19)	143 ± 5^b,e^	3.6 ± 0.4^b,e^	99 ± 7^b^	0.96 ± 0.45^d^	134 ± 123
24 hours					
Control (*n* = 19)	138 ± 5	4.3 ± 0.5	104 ± 4	1.30 ± 0.49	168 ± 133
Lactate (*n* = 18)	146 ± 6^b,e^	3.3 ± 0.4^b,e^	98 ± 9^b^	0.98 ± 0.40^d^	136 ± 128
48 hours					
Control (*n* = 18)	138 ± 7	4.3 ± 0.4	102 ± 4	1.22 ± 0.33	156 ± 130
Lactate (*n* = 18)	144 ± 6^b,e^	3.8 ± 0.5	103 ± 6	1.15 ± 0.41	129 ± 119

^a^Cl - = Plasma chloride concentration (mmol/L), Crea = Plasma creatinine concentration (μmol/L), K^+^ = Plasma potassium concentration (mmol/L), Na^+^ = Plasma sodium concentration (mmol/L), P = Plasma phosphate concentration (mmol/L). ^b^*P* < 0.01 compared to baseline, ^c^*P* < 0.05, ^d^*P* < 0.05, ^e^*P* < 0.01 intergroup comparison. Data are expressed as mean ± SD.

### Fluid balance

The fluid balance in the first 24 hours was 1,296 ± 1,633 ml in the lactate group versus 845 ± 1,421 ml in the control group. The fluid balance was -339 ± 1,453 ml in the controls and 230 ± 991 ml in the lactate group from 24 to 48 hours. Once adjusted for the volume of study fluid, the mean fluid balance was 586 ml in the control group and -843 ml in the lactate group at 24 hours, and it was -598 ml and -1,909 ml in the control and lactate groups, respectively, at 48 hours. We found no correlation between fluid balance and the change in SV in the lactate group and a slightly negative correlation in the control group at 24 hours. After adjusting for fluid balance by ANCOVA, the results still showed strong support for a treatment effect of lactate, with the lactate group exhibiting an estimated CO of 0.91 L/min more, on average, than the control group (*P* = 0.011) (Table 3).

### Other organ function, length of stay and mortality

There were no significant differences in other organ functions between the groups, although serum creatinine (Table 4) tended to be lower in the lactate group. Likewise, there was no significant difference between the groups regarding the ICU and hospital length of stay or 28-day mortality (five in the lactate group vs. four in the control group) or 90-day mortality (five in each group).

## Discussion

The main finding of this randomised controlled trial is that hypertonic half-molar sodium lactate infusion (3 ml/kg over 15 minutes followed by 1 ml/kg/h for 24 hours) increased CO in critically ill patients with AHF. The effect was related to a significant increase in SV rather than HR. We also observed improvement in right ventricular systolic function as assessed by TAPSE. The infusion of half-molar sodium lactate was safe and well tolerated, but it had a marked effect on acid-base status and plasma electrolytes. The total amount of intravenous sodium lactate (13.5 mmol/kg/24 h, which is roughly equal to three-fourths of endogenous daily lactate production in resting humans) was associated with modest metabolic alkalosis and hypernatraemia as well as mild hypochloraemia, hypokalaemia and hypophosphataemia.

Under normal conditions, the main energy supply for the heart is the oxidation of fatty acids, whereas other nutrients such as glucose lactate and amino acids contribute to myocardial ATP generation in varying proportions. At rest, approximately 10% to 40% of energy is derived from pyruvate formed either by glycolysis or the conversion of lactate. Although fatty acids have higher yields of ATP per molecule, the ATP yield per oxygen molecule utilized is 5% to 10% better with lactate and glucose, respectively [21]. During exercise, β-adrenergic stimulation, elevated afterload and fast pacing, both lactate uptake by the myocardium and its use as metabolic fuel increase [17,22-24]. Lactate may even exceed glucose as an oxidative substrate in the presence of elevated plasma lactate levels [21]. In rats with ischaemia-induced chronic heart failure, maximum lactate influx into isolated cardiomyocytes was increased by 250% and the lactate transporter MCT1 protein level was increased by 260%, suggesting increased utilization of lactate [25]. Of relevance is that sodium lactate infusion has been reported to increase CO in anaesthetized pigs after surgery, whereas whole-body oxygen utilization tended to increase, suggesting lactate oxidation [26]. Studies in patients have demonstrated that exogenous half-molar sodium lactate infusion is well tolerated after major elective surgery and increases CO in patients after cardiac bypass surgery [27,28].

Although we do not provide direct evidence of lactate oxidation by the heart, the relatively low plasma lactate levels, together with an increase in SV and CO in the lactate group, suggest myocardial lactate metabolism. Previous studies have documented lactate oxidation in patients with cardiogenic shock while receiving a short-term sodium lactate infusion (2.5 mmol/kg for 15 minutes) [17]. The effects of sodium lactate on acid-base balance and electrolytes in our group of critically ill patients with AHF also suggest increased lactate metabolism. Others have reported an increased rate of gluconeogenesis and lactate oxidation during sodium lactate infusion [16,18]. Nevertheless, the effects of sodium lactate on CO may not be fully attributable to lactate oxidation by the myocardium as the resulting metabolic alkalosis *per se* may lead to improved myocardial function [28]. As the lactate anion is metabolized in the cells, the sodium is expelled, thus increasing strong ion difference in the interstitial space and plasma and causing metabolic alkalosis according to the Stewart concept [18,27,29]. However, alkalosis has an effect on systemic vascular resistance and endogenous production of catecholamines, making the effect of alkalosis on CO variable, and we believe smaller, compared to the utilization of lactate [30-32]. Nevertheless, in our present study, it was impossible to distinguish between the contribution of sodium lactate metabolism and resulting metabolic alkalosis because the latter follows the former. It is unlikely that hypernatraemia, hypokalaemia or hypophosphataemia led to the marked increase in SV, TAPSE and CO in our patients with AHF.

### Limitations

We conducted a pilot, single-centre, open-label clinical trial. Despite the relatively small number of randomised patients, we demonstrate a significant effect of half-molar sodium lactate on cardiac function, a finding similar those reported by others [18,26]. We did not utilize continuous measurement of CO in our study, because this is not a universal practice in our ICU. Instead, we used comprehensive echocardiographic assessment by skilled operators, and a qualified reporter blinded to treatment group assignment measured the echocardiographic parameters. An important limitation of this study is the imbalance in the amount of fluid infusion between the two groups, resulting in possible confounding effects of positive fluid balance. However, the ANCOVA shows that, after adjusting for the nonsignificant baseline imbalance in CO and the fluid balance over the course of 24 hours, CO still significantly increased in the group that received lactate. Despite the increase in CO, there was no effect on length of ICU or hospital stay, mechanical ventilation or survival; however, the study was not sufficiently powered for those endpoints.

## Conclusions

The findings of our present study regarding increased SV, CO and right ventricular systolic function in critically ill patients with AHF receiving half-molar sodium lactate challenge the dogma that exogenous lactate is detrimental to myocardial function in the critically ill and support the view that endogenous lactate may be an important substrate for myocardium during periods of physiological stress [33]. We suggest that infusion of half-molar sodium lactate may improve CO in AHF patients with reduced LVEF; however, its clinical use may be limited in patients with alkalaemia or hypernatraemia or in those with severely compromised liver and renal function.

## Key messages

• Infusion of half-molar sodium lactate (13.5 mmol over 24 hours) improved cardiac performance, challenging the dogma that lactate *per se* is detrimental to myocardial function.

• Our results support an important role of lactate in cardiac metabolism during periods of critical illness.

• Half-molar sodium lactate can be thought of as an alternative inotrope in AHF patients with reduced LVEF who possess organ function sufficient for lactate metabolism.

## Competing interests

XML was a member of the Innogene International Scientific Board of Advisors. The other authors declare that they have no competing interests.

## Authors’ contributions

MN conceived of and designed the study, collected and analysed the data and was involved with manuscript writing. XL conceived of and designed the study, was responsible for logistics and drafted the manuscript. SH was involved with the study design, data analysis and manuscript writing. LW and IT were involved with the study design, data collection and analysis and manuscript writing. RP collected and analysed data and was involved with writing the manuscript. IMS was involved in the study design, data collection and manuscript writing. ASM obtained financial support, collected and analysed the data and was involved with manuscript writing. All authors, except the late Prof Xavier Maurice Leverve, read and approved the final manuscript.
